# Alcohol Use Disorder Polygenic Score Compared With Family History and *ADH1B*

**DOI:** 10.1001/jamanetworkopen.2024.52705

**Published:** 2024-12-30

**Authors:** Dongbing Lai, Michael Zhang, Marco Abreu, Tae-Hwi Schwantes-An, Grace Chan, Danielle M. Dick, Chella Kamarajan, Weipeng Kuang, John I. Nurnberger, Martin H. Plawecki, John Rice, Marc Schuckit, Bernice Porjesz, Yunlong Liu, Tatiana Foroud

**Affiliations:** 1 Department of Medical and Molecular Genetics, Indiana University School of Medicine, Indianapolis; 2 Department of Psychiatry, University of Connecticut School of Medicine, Farmington; 3 Department of Psychiatry, Roy J. and Lucille A. Carver College of Medicine, University of Iowa, Iowa City; 4 Department of Psychiatry, Robert Wood Johnson Medical School, Rutgers University, Piscataway, New Jersey; 5 Henri Begleiter Neurodynamics Laboratory, Department of Psychiatry, SUNY Downstate Health Science University, New York, New York; 6 Department of Psychiatry, Indiana University School of Medicine, Indianapolis; 7 Department of Psychiatry, Washington University in St Louis School of Medicine, St Louis, Missouri; 8 Department of Psychiatry, University of California San Diego Medical School, San Diego

## Abstract

**Importance:**

Identification of individuals at high risk of alcohol use disorder (AUD) and subsequent application of prevention and intervention programs has been reported to decrease the incidence of AUD. The polygenic score (PGS), which measures an individual’s genetic liability to a disease, can potentially be used to evaluate AUD risk.

**Objective:**

To assess the estimability and generalizability of the PGS, compared with family history and *ADH1B*, in evaluating the risk of AUD among populations of European ancestry.

**Design, Setting, and Participants:**

This genetic association study was conducted between October 1, 2023, and May 21, 2024. A 2-stage design was used. First, the pruning and thresholding method was used to calculate PGSs in the screening stage. Second, the estimability and generalizability of the best PGS was determined using 2 independent samples in the testing stage. Three cohorts ascertained to study AUD were used in the screening stage: the Collaborative Study on the Genetics of Alcoholism (COGA), the Study of Addiction: Genetics and Environment (SAGE), and the Australian Twin-Family Study of Alcohol Use Disorder (OZALC). The All of Us Research Program (AOU), which comprises participants with diverse backgrounds and conditions, and the Indiana Biobank (IB), consisting of Indiana University Health system patients, were used to test the best PGS. For the COGA, SAGE, and OZALC cohorts, cases with AUD were determined using *Diagnostic and Statistical Manual of Mental Disorders, Fourth Edition* (*DSM-IV*) or *Fifth Edition* (*DSM-5*) criteria; controls did not meet any criteria or did not have any other substance use disorders. For the AOU and IB cohorts, cases with AUD were identified using *International Classification of Diseases, Ninth Revision* (*ICD-9*) or *International Classification of Diseases, Tenth Revision* (*ICD-10*) codes; controls were aged 21 years or older and did not have AUD.

**Exposure:**

The PGS was calculated using single-nucleotide variants with concordant effects in 3 large-scale genome-wide association studies of AUD-related phenotypes.

**Main Outcomes and Measures:**

The main outcome was AUD determined with *DSM-IV* or *DSM-5* criteria and *ICD-9* or *ICD-10* codes. Generalized linear mixed models and logistic regression models were used to analyze related and unrelated samples, respectively.

**Results:**

The COGA, SAGE, and OZALC cohorts included a total of 8799 samples (6323 cases and 2476 controls; 50.6% were men). The AOU cohort had a total of 116 064 samples (5660 cases and 110 404 controls; 60.4% were women). The IB cohort had 6373 samples (936 cases and 5437 controls; 54.9% were women). The 5% of samples with the highest PGS in the AOU and IB cohorts were approximately 2 times more likely to develop AUD (odds ratio [OR], 1.96 [95% CI, 1.78-2.16]; *P* = 4.10 × 10^−43^; and OR, 2.07 [95% CI, 1.59-2.71]; *P* = 9.15 × 10^−8^, respectively) compared with the remaining 95% of samples; these ORs were comparable to family history of AUD. For the 5% of samples with the lowest PGS in the AOU and IB cohorts, the risk of AUD development was approximately half (OR, 0.53 [95% CI, 0.45-0.62]; *P* = 6.98 × 10^−15^; and OR, 0.57 [95% CI, 0.39-0.84]; *P* = 4.88 × 10^−3^) compared with the remaining 95% of samples; these ORs were comparable to the protective effect of *ADH1B*. PGS had similar estimabilities in male and female individuals.

**Conclusions and Relevance:**

In this study of AUD risk among populations of European ancestry, PGSs were calculated using concordant single-nucleotide variants and the best PGS was tested in targeted datasets. The findings suggest that the PGS may potentially be used to evaluate AUD risk. More datasets with similar AUD prevalence as in general populations are needed to further test the generalizability of PGS.

## Key Points

**Question:**

Can a polygenic score (PGS) be used to evaluate the risk of alcohol use disorder (AUD) among populations of European ancestry?

**Findings:**

In this genetic association study, a PGS was derived using single-nucleotide variants with concordant effects in different study cohorts. In 2 independent datasets, the top 5% of samples with the highest PGS were approximately 2 times more likely to develop AUD compared with the remaining 95% of samples; for the bottom 5% of samples with the lowest PGS, the risk of AUD development was approximately half.

**Meaning:**

These findings suggest that a PGS calculated using concordant single-nucleotide variants may potentially be used to evaluate AUD risk.

## Teaser

This genetic association study evaluates the estimability and generalizability of a polygenic score, compared with family history and *ADH1B*, in assessing the risk of alcohol use disorder among populations of European ancestry.

## Introduction

Alcohol use disorder (AUD), characterized by excessive and uncontrolled alcohol consumption despite adverse social, mental, and health consequences, represents a substantial public health challenge.^1^ In 2021, 11.2% of US adults and, remarkably, 2.9% of youths aged 12 to 17 years had AUD.^2^ Beyond the direct consequences of AUD on mental and physical health, individuals with the disorder have increased risk of more than 200 diseases^1^ and 4.7 times greater risk of mortality.^3^ Moreover, adverse social and mental outcomes have been observed for family members of individuals with AUD, especially children, which can exert a lasting effect on the health of future generations (eg, fetal alcohol spectrum disorders).

AUD is preventable. Effective and efficient prevention of AUD lies in identifying high-risk individuals and then applying prevention and intervention programs promptly.^4,5,6,7,8,9,10,11,12^ Commonly used screening methods, such as the Alcohol Use Disorders Identification Test (AUDIT),^13^ focus on drinking patterns and alcohol-related problems; therefore, they are designed for individuals who have already started drinking, and they overlook those who currently do not drink or do not yet exhibit alcohol-related problems but could rapidly progress. Furthermore, underage drinkers may not accurately report their drinking history; this is particularly crucial for adolescents and young adults, because alcohol can cause substantial harm to their neural development. Additionally, for many individuals, waiting for demonstration of alcohol-related problems means that they may have already progressed beyond the efficacy of some prevention and intervention programs. Therefore, identifying individuals at high risk of AUD before they start drinking can minimize the potential harm of alcohol consumption and maximize the effectiveness of prevention and intervention programs.

Family history of AUD does not rely on an individual’s drinking history^14,15,16,17^ and has high accuracy to identify individuals at high risk^18^; however, not all individuals know their family history.^19^ Importantly, for polygenic disorders such as AUD, many affected individuals are not expected to have a positive family history based on the polygenic theory.^20,21,22^ In fact, in a US national survey, approximately 50% of males and 43% of females with AUD did not have a family history of AUD.^23^ The estimated heritability of AUD is approximately 50%; therefore, genetic factors may potentially be used to identify high-risk individuals as a complementary tool for those do not know or do not report family history. Additionally, previous studies have reported that the genetic risk of AUD was also related to AUD severity^24^ and remission from AUD.^25^ Therefore, identifying individuals at high or low risk of AUD may help elucidate the mechanism of AUD and facilitate the development of personalized prevention and intervention and treatment programs.

A polygenic score (PGS) is the weighted sum of single-nucleotide variant (SNV) risk alleles across the entire genome and has shown promise in evaluating disease risks.^26,27,28,29,30,31,32,33^ SNVs and their weights are derived from discovery datasets, and PGSs are calculated and tested in target datasets. In our previous work, we used concordant SNV (ie, SNVs with the same directions of effects in different study cohorts or populations) strategies to calculate PGSs.^24,34,35^ These strategies exclude large numbers of irrelevant SNVs while retaining disease-associated SNVs, thereby substantially increasing the estimability of PGSs. In a population of European ancestry, PGS estimability was comparable to family history of AUD and was associated with AUD severity and remission from AUD.^24^ However, in that study, PGS was calculated and tested in a family cohort primarily ascertained for individuals with AUD.^24^

This study aimed to further increase PGS estimability and test PGS generalizability in populations of European ancestry. We optimized a PGS workflow by using a 2-stage design and included more discovery datasets and target datasets ascertained for different purposes to calculate PGSs and test the best PGS.

## Methods

This genetic association study was approved by the institutional review boards of all participating sites (Indiana University School of Medicine, SUNY Downstate Health Science University, Washington University School of Medicine, University of Connecticut School of Medicine, University of Iowa Roy J. and Lucille A. Carver College of Medicine, and University of California San Diego Medical School). All participants provided written consent. The study followed the Strengthening the Reporting of Genetic Association Studies (STREGA) reporting guideline.

### Study Overview

This study was conducted between October 1, 2023, and May 21, 2024. The study design is shown in the Figure. We chose the 2-stage design by following the pioneering work of Khera et al,^29,30^ who developed the first potentially clinically applicable PGS. Multiple PGSs were tested in the screening stage and then the PGS with the highest estimability moved to the testing stage, avoiding multiple testing problems and reducing computational burdens. In this study, we calculated multiple PGSs by using different sets of concordant SNVs in the screening stage. Concordant SNVs were identified from 3 large-scale genome-wide association studies (GWASs) of AUD-related phenotypes: the Million Veteran Program (MVP),^36^ the UK Biobank (UKBB),^37^ and the FinnGen Consortium (FinnGen).^38^ The Collaborative Study on the Genetics of Alcoholism (COGA) and 2 datasets from the Database of Genotypes and Phenotypes (dbGaP) (phs000092.v1.p1 from the Study of Addiction: Genetics and Environment [SAGE], and phs000181.v1.p1 from the Australian Twin-Family Study of Alcohol Use Disorder [OZALC]) were used as the screening datasets. Two independent datasets, the All of Us Research Program (AOU) and the Indiana Biobank (IB),^25,34^ were used as the testing datasets. The AOU comprises participants with diverse backgrounds and conditions, and the IB consists of Indiana University Health system patients.

**Figure.**
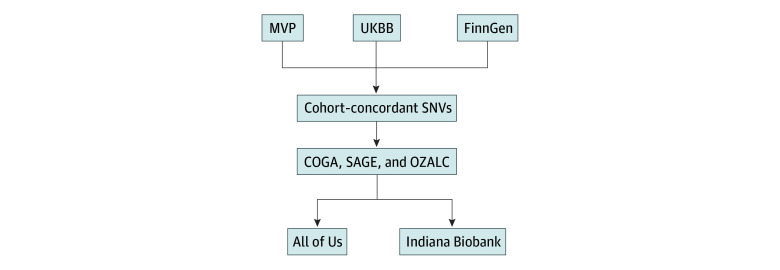
Study Flow Diagram COGA indicates Collaborative Study on the Genetics of Alcoholism; FinnGen, FinnGen Consortium; MVP, Million Veteran Program; OZALC, Australian Twin-Family Study of Alcohol Use Disorder; SAGE, Study of Addiction: Genetics and Environment; SNV, single-nucleotide variant; UKBB, UK Biobank.

### Discovery Datasets and Meta-Analysis

GWAS summary statistics for the MVP dataset were downloaded from the dbGaP (phs001672) (N = 202 004, with 34 658 cases and 167 346 controls). FinnGen release r9 (N = 377 277, with 15 715 cases and 361 562 controls) was downloaded from the FinnGen study site.^39^ The AUD status of samples in the MVP and FinnGen datasets was determined using *International Classification of Diseases, Ninth Revision* (*ICD-9*) or *International Classification of Diseases, Tenth Revision* (*ICD-10*) codes. For the UKBB dataset, the GWAS of the AUDIT problem subscale (N = 121 604) was provided by the authors.^37^ All 3 GWASs were limited to populations of European ancestry. Palindromic SNVs were excluded to avoid strand ambiguity. Meta-analysis was performed to estimate SNV effects by using METAL software (University of Michigan) weighted by effective sample sizes, which were calculated as follows: 4/(1/No. of cases + 1/No. of controls).^40,41^ We used effective sample sizes due to the small percentages of cases in these cohorts. For the UKBB dataset, we used an AUDIT score of 12 or greater (range, 0-40) to determine the number of cases for the purpose of calculating the effective sample size as suggested by the authors. Only SNVs with the same directions of effects in all 3 GWASs as determined with METAL software^40^ were kept.

### Screening and Testing Datasets

Accurate diagnosis of cases and controls is crucial for screening PGS. Therefore, we used 3 datasets from cohorts ascertained to study AUD (COGA, SAGE, and OZALC). AUD was determined using *Diagnostic and Statistical Manual of Mental Disorders, Fifth Edition* (*DSM-5*) criteria in the COGA cohort and *DSM, Fourth Edition* (*DSM-IV*) criteria in the SAGE and OZALC cohorts. Those who did not meet any *DSM-IV* or *DSM-5* criteria were considered controls. Additionally, because AUD is substantially genetically correlated with other substance use disorders, we also excluded those with other substance use disorders from the controls. These 3 datasets were combined as the screening dataset.

Samples of populations with European ancestry from the AOU, version 7, and the IB datasets^25,34^ were used as testing datasets. The AOU is a national research resource with participants from diverse backgrounds and conditions.^42^ The IB is a statewide collaboration that provides centralized processing and storage of specimens that are linked to participants’ electronic medical information. For both the AOU and IB cohorts, AUD status was determined based on *ICD-9* or *ICD-10* codes. Those who were aged 21 years or older and without AUD status were considered as controls. We did not exclude those having other substance use disorders from the controls, due to the large numbers of controls and the small prevalence of AUD and substance use disorders in both the AOU and IB cohorts.

Detailed screening and testing data processing and imputation information are provided in the eMethods in Supplement 1. Concordant SNVs that passed quality control in all datasets were used to ensure that the same set of SNVs was used in calculating the PGS in different datasets.

### PGS Calculation

We used the pruning and thresholding method because it can select SNVs that have the largest contributions for the purpose of developing a specific array for AUD PGS calculation to eliminate genotyping platform effects. First, PLINK, version 2,^43,44^ was used to select sets of independent concordant SNVs by varying *P* value thresholds (>.99, .50, .20, .10, .05, .01, .005, .001, .0005, .0001, .00005,.00001, .000005, .000001, .0000005, .0000001, and .00000005), linkage disequilibrium (LD) *r*^2^ values (0.1, 0.2, 0.3, 0.4, and 0.5), and physical distance to calculate *r*^2^ (250 kb and 500 kb), resulting in 170 sets of SNVs. LD was determined using 1000 Genomes Project samples of European ancestry. For individual *i*, PGS was calculated as PGS*i* = Σ*^M^*_(_*_j_*_=1)_ β*_j_* × dosage*_ij_*, where *M* is the number of SNVs and β*_j_* is the *z* score for SNV *j* estimated from meta-analysis of GWAS of MVP, UKBB, and FinnGen; dosage*_ij_* is the number of effective alleles in the AOU dataset and imputation dosage in the IB dataset for individual *i* for SNV *j*.

### Statistical Analysis

One of the major goals of the PGS is to identify individuals at high or low risk. Therefore, we dichotomized PGSs as high or low risk using different thresholds. To determine the best thresholds, we defined high-risk groups as the top 5% to 50% (in increments of 5%) of samples with the highest PGS, and we defined low-risk groups as the bottom 5% to 45% (in increments of 5%) of samples with the lowest PGS. Then each high-risk or low-risk group was compared with the remaining samples; for example, the top 5% of samples were compared with the remaining 95%. The PGS distributions were determined in the screening dataset (COGA, SAGE, and OZALC combined), the AOU dataset, and the IB dataset separately. COGA and OZALC were familial cohorts, and some SAGE samples were related. Therefore, we fit generalized linear mixed models using generalized estimating equations; specifically, we included a random intercept to adjust for the family relationships. Unrelated samples from the AOU and IB datasets were included; thus, we used logistic regression. Sex, the first 10 principal components of genetic ancestries, and genotyping arrays in each dataset were included as covariates. These datasets used different definitions of age; thus, age was not included as a covariate, as in a previous large AUD GWAS.^45^ SAS, version 9.4 (SAS Institute Inc), was used to perform all statistical analyses. Additionally, for the best PGS, we tested estimabilities in male and female individuals separately. Because the rs1229984 T allele in *ADH1B* is the largest protective genetic factor for AUD in populations of European ancestry,^46^ we also tested the best PGS by excluding carriers of the rs1229984 T allele.

## Results

The COGA, SAGE, and OZALC cohorts included a total of 8799 samples (6323 cases and 2476 controls; 50.6% were men and 49.4% were women). The AOU cohort had a total of 116 064 samples (5660 cases and 110 404 controls; 39.6% were men and 60.4% were women). The IB cohort had 6373 samples (936 cases and 5437 controls; 45.1% were men and 54.9% were women). A summary of the screening and testing datasets is presented in Table 1. The number of controls was much smaller than the number of cases in the screening datasets due to the definition of controls. The majority of AOU and IB samples were classified as controls.

**Table 1.** Summary of the Target Datasets

Stage and dataset	Total No. (%) of samples		No. (%) of cases		No. (%) of controls	
	Men	Women	Men	Women	Men	Women
Screen						
COGA (n = 5655)	2791 (49.4)	2864 (50.6)	2340 (57.9)	1703 (41.1)	451 (28.0)	1161 (72.0)
SAGE (n = 967)	429 (44.4)	538 (55.6)	332 (52.7)	298 (47.3)	97 (28.8)	240 (71.2)
OZALC (n = 2177)	1236 (56.8)	941 (43.2)	1027 (62.2)	623 (37.8)	209 (39.7)	318 (60.3)
Total (n = 8799)	4456 (50.6)	4343 (49.4)	3699 (58.5)	2,624 (41.5)	757 (30.6)	1719 (69.4)
Test						
AOU (n = 116 064)	45 928 (39.6)	70 136 (60.4)	3535 (62.5)	2125 (37.5)	42 393 (38.4)	68 011 (61.6)
IB (n = 6373)	2875 (45.1)	3498 (54.9)	548 (58.6)	388 (41.4)	2327 (42.8)	3110 (57.2)
Total (n = 112 437)	48 803 (39.9)	73 634 (60.1)	4083 (61.9)	2513 (38.1)	44 720 (38.6)	71 121 (61.4)

Abbreviations: AOU, All of Us Research Program; COGA, Collaborative Study on the Genetics of Alcoholism; IB, Indiana Biobank; OZALC, Australian Twin-Family Study of Alcohol Use Disorder; SAGE, Study of Addiction: Genetics and Environment.

eTable 1 in Supplement 2 presents the results of each PGS analysis in the screening stage. For the high-risk analysis, the best PGS was calculated using 9154 SNVs having *P* < .01 and LD *r*^2^ < 0.1 within 250 kb when comparing the top 5% of samples with the remaining 95% (odds ratio [OR], 2.75 [95% CI, 2.05-3.71]; *P* = 2.34 × 10^−11^). For the low-risk analysis, the best PGS was calculated using 99 135 SNVs having *P* < .50 and LD *r*^2^ < 0.2 within 500 kb when comparing the bottom 25% of samples with the remaining 75% (OR, 0.53 [95% CI, 0.47-0.60]; *P* = 2.88 × 10^−26^). However, using 9154 SNVs provided the best result in the high-risk group, had a comparable result in the low-risk group (for the bottom 45%: OR, 0.61 [95% CI, 0.55-0.67]) (eTable 2 in Supplement 2), and used a smaller number of SNVs; therefore, this PGS was used in the testing stage. The list of 9154 SNVs and their weights is presented in eTable 2 in Supplement 2.

The results of testing the best PGS in the AOU and IB cohorts are presented in Table 2. All high-risk or low-risk groups were associated with AUD when compared with the remaining samples. The top 5% of samples had the highest risk in both the AOU (OR, 1.96 [95% CI, 1.78-2.16]) and IB (OR, 2.07 [95% CI, 1.59-2.71]) cohorts, and they were 2 times more likely to develop AUD. The risk of AUD development was approximately half for samples in the bottom percentages of risk in the AOU and IB cohorts: The bottom 10% had the lowest risk in the AOU cohort (OR, 0.52 [95% CI, 0.46-0.58]), whereas the bottom 5% had the lowest risk in the IB cohort (OR, 0.57 [95% CI, 0.39-0.84]). However, the bottom 5% in the AOU cohort had the second lowest risk (OR, 0.53 [95% CI, 0.45-0.62]); therefore, we used the bottom 5% in sex-stratified analysis with results presented in Table 3. All PGSs were associated with AUD except the bottom 5% in females in the IB cohort. Overall, males and females had similar results.

**Table 2.** Results of PGS Analysis in Testing Datasets

PGS threshold	OR (95% CI)	β (SE)	*P* value	No. in the high-risk or low-risk group		No. in the remaining sample	
				Cases	Controls	Cases	Controls
AOU dataset							
Top, %							
5	1.96 (1.78-2.16)	0.67 (0.05)	4.10 × 10^−43^	510	5294	5150	105 110
10	1.88 (1.75-2.02)	0.63 (0.04)	9.19 × 10^−64^	943	10 664	4717	99 740
15	1.75 (1.64-1.87)	0.56 (0.03)	6.53 × 10^−65^	1304	16 106	4356	94 298
20	1.74 (1.64-1.85)	0.56 (0.03)	4.95 × 10^−75^	1681	21 532	3979	88 872
25	1.72 (1.63-1.82)	0.54 (0.03)	2.70 × 10^−79^	2024	26 992	3636	83 412
30	1.69 (1.60-1.78)	0.52 (0.03)	7.73 × 10^−78^	2333	32 486	3327	77 918
35	1.71 (1.62-1.81)	0.54 (0.03)	7.28 × 10^−85^	2669	37 954	2991	72 450
40	1.69 (1.60-1.78)	0.52 (0.03)	9.47 × 10^−81^	2953	43 473	2707	66 931
45	1.68 (1.59-1.77)	0.52 (0.03)	9.58 × 10^−78^	3233	48 996	2427	61 408
50	1.68 (1.59-1.77)	0.52 (0.03)	4.66 × 10^−75^	3505	54 527	2155	55 877
Bottom, %							
45	0.60 (0.57-0.64)	−0.51 (0.03)	5.17 × 10^−69^	1906	50 323	3754	60 081
40	0.60 (0.56-0.63)	−0.52 (0.03)	2.14 × 10^−66^	1646	44 780	4014	65 624
35	0.58 (0.55-0.62)	−0.54 (0.03)	1.90 × 10^−64^	1387	39 236	4273	71 168
30	0.56 (0.53-0.60)	−0.57 (0.03)	1.53 × 10^−63^	1132	33 687	4528	76 717
25	0.58 (0.54-0.62)	−0.55 (0.04)	5.16 × 10^−51^	939	28 077	4721	82 327
20	0.58 (0.54-0.63)	−0.54 (0.04)	2.22 × 10^−41^	737	22 476	4923	87 928
15	0.56 (0.51-0.61)	−0.58 (0.05)	1.04 × 10^−35^	525	16 885	5135	93 519
10	0.52 (0.46-0.58)	−0.66 (0.06)	3.11 × 10^−29^	320	11 287	5340	99 117
5	0.53 (0.45-0.62)	−0.64 (0.08)	6.98 × 10^−15^	160	5644	5500	104 760
IB dataset							
Top, %							
5	2.07 (1.59-2.71)	0.73 (0.14)	9.15 × 10^−8^	80	239	856	5198
10	1.70 (1.39-2.10)	0.53 (0.11)	4.36 × 10^−7^	135	503	801	4934
15	1.61 (1.35-1.92)	0.48 (0.09)	1.53 × 10^−7^	193	763	743	4674
20	1.51 (1.28-1.78)	0.41 (0.08)	7.70 × 10^−7^	243	1032	693	4405
25	1.55 (1.33-1.80)	0.44 (0.08)	2.07 × 10^−8^	303	1291	633	4146
30	1.56 (1.35-1.81)	0.45 (0.07)	1.82 × 10^−9^	360	1552	576	3885
35	1.55 (1.34-1.79)	0.44 (0.07)	1.85 × 10^−9^	409	1822	527	3615
40	1.54 (1.34-1.77)	0.43 (0.07)	1.78 × 10^−9^	459	2091	477	3346
45	1.52 (1.32-1.75)	0.42 (0.07)	6.48 × 10^−9^	504	2364	432	3073
50	1.62 (1.40-1.86)	0.48 (0.07)	4.48 × 10^−11^	562	2625	374	2812
Bottom, %							
45	0.66 (0.57-0.76)	−0.42 (0.07)	1.68 × 10^−8^	340	2527	596	2910
40	0.67 (0.58-0.78)	−0.40 (0.08)	1.10 × 10^−7^	300	2249	636	3188
35	0.70 (0.60-0.82)	−0.35 (0.08)	8.25 × 10^−6^	266	1964	670	3473
30	0.69 (0.59-0.81)	−0.37 (0.08)	8.18 × 10^−6^	221	1690	715	3747
25	0.70 (0.59-0.83)	−0.36 (0.09)	5.46 × 10^−5^	182	1411	754	4026
20	0.72 (0.60-0.87)	−0.33 (0.10)	7.58 × 10^−4^	147	1127	789	4310
15	0.66 (0.53-0.83)	−0.41 (0.11)	2.48 × 10^−4^	102	853	834	4584
10	0.60 (0.45-0.78)	−0.52 (0.14)	2.28 × 10^−4^	61	576	875	4861
5	0.57 (0.39-0.84)	−0.56 (0.20)	4.88 × 10^−3^	29	289	907	5148

Abbreviations: AOU, All of Us Research Program; IB, Indiana Biobank; OR, odds ratio; PGS, polygenic score.

**Table 3.** Results of PGS Analysis for Males and Females

Sex	AUD prevalence, %	PGS threshold, %	OR (95% CI)	β (SE)	*P* value	No. in the high-risk or low-risk group		No. in the remaining sample	
						Cases	Controls	Cases	Controls
AOU dataset									
Female	3.19	Top 5	1.97 (1.69-2.30)	0.68 (0.08)	1.81 × 10^−18^	322	5619	3529	111 222
Male	8.53	Top 5	1.96 (1.73-2.22)	0.67 (0.06)	2.45 × 10^−26^	527	3532	6234	68 978
Female	3.19	Bottom 5	0.60 (0.47-0.77)	−0.51 (0.13)	6.36 × 10^−5^	131	5865	3720	110 976
Male	8.53	Bottom 5	0.49 (0.40-0.60)	−0.72 (0.11)	1.60 × 10^−11^	222	3782	6539	68 728
IB dataset									
Female	11.09	Top 5	2.14 (1.45-3.15)	0.76 (0.20)	1.35 × 10^−4^	35	141	353	2969
Male	19.06	Top 5	2.02 (1.40-2.93)	0.70 (0.19)	1.82 × 10^−4^	45	98	503	2229
Female	11.09	Bottom 5	0.67 (0.38-1.18)	−0.40 (0.29)	.16	14	162	374	2948
Male	19.06	Bottom 5	0.49 (0.29-0.85)	−0.71 (0.28)	.01	15	127	533	2200

Abbreviations: AOU, All of Us Research Program; IB, Indiana Biobank; OR, odds ratio; PGS, polygenic score.

There were 9413 carriers of the rs1229984 T allele in the AOU cohort (229 cases and 9184 controls) and 369 in the IB cohort (47 cases and 322 controls). When they were excluded from analysis, the results were similar (eTable 3 in Supplement 2), possibly due to the small numbers of carriers.

## Discussion

In this study, we calculated the AUD PGS using concordant SNVs and a 2-stage design. In the AOU and IB cohorts (which were not ascertained to study AUD), the top 5% with the highest PGS were 2 times more likely to develop AUD (ORs of 1.96 and 2.07) compared with the remaining 95%, whereas the risk was approximately half for the bottom 5% (ORs of 0.53 and 0.57). The PGS had similar estimabilities in both male and female individuals.

Dataset properties have great importance in terms of PGS estimability and generalizability. The choice of dataset is especially crucial when the objective is to identify high-risk and low-risk individuals from general populations. Cohorts ascertained to study AUD aim to recruit more cases; thus, large percentages of participants have a higher PGS. Consequently, the PGS exhibits high estimability within these cohorts but lower estimability in less specially recruited cohorts. Additionally, because many of these cohorts aim to recruit controls that are otherwise similar to cases, many controls likely exhibit alcohol use problems but they are not severe enough for an AUD diagnosis. Furthermore, some controls do not have AUD but may be experiencing other substance use disorders. Including these individuals as controls reduces estimability of the PGS. In our study, we used AUD cohorts for screening. AUD status was determined using *DSM-IV* or *DSM-5* criteria, hence enhancing diagnostic precision. Moreover, we excluded individuals with alcohol use problems but not meeting AUD criteria and other substance use disorders from the control group, thereby increasing statistical power. All of these approaches maximize our ability to screen for PGS with the highest estimability. Then we tested the generalizability of the best PGS in the AOU and IB cohorts, which were not ascertained to study AUD. The large sample sizes and low prevalence of AUD in both the AOU and IB cohorts ensured that PGS distributions remained unbiased and may resemble those in general populations. The ORs for the AOU and IB cohorts were 1.96 and 2.07, respectively, which were comparable to those for a family history of AUD (OR, 1.91-2.38).^14,47^ We note that the estimability is smaller than the PGS of some diseases; however, for preventable disorders like AUD, the estimabilities of the PGS could be totally reduced or amplified by nongenetic factors (eg, culture or religion can prevent individuals with high PGS from drinking, whereas traumatic events can increase the probability of AUD for those with low PGS). Consequently, higher PGS estimability is not expected for preventable diseases. It is noteworthy that the aforementioned ORs for both the AOU and IB cohorts (approximately 2) were near the suggested threshold for a PGS to be incorporated in clinical settings to provide additional information for risk evaluation.^48,49^

Although female individuals have substantially lower AUD prevalence than male individuals,^2^ the PGS had similar results for both sexes in this study (Table 3). Therefore, this suggests that the different prevalence may be due to nongenetic factors. Studies have found that sex differences of AUD and related problems have decreased substantially in the US.^50^ Because both sexes show similar genetic risks for AUD development, identifying which drinking-related behaviors have changed in female individuals could shed light on the etiologies of AUD and mitigate AUD and related issues.

As noted earlier, the risk of AUD development was approximately half (ORs of 0.53 and 0.57, respectively) for the bottom 5% of samples with the lowest PGS in the AOU and IB cohorts. These ORs were similar to the OR for rs1229984 estimated from the MVP dataset^36^ (OR, 0.57 [95% CI, 0.54-0.61]) and close to the OR estimated from the FinnGen dataset^38^ (OR, 0.41 [95% CI, 0.33-0.51]). The rs1229984 T allele frequencies were 4.2% in the AOU dataset, 2.9% in the IB dataset, 3.0% in the MVP dataset, and 0.5% in the FinnGen dataset. The low allele frequency may be the reason for the smaller OR in the FinnGen dataset due to the smaller number of T allele carriers. We did not include rs1229984 in our calculations of PGS because it did not pass the Hardy-Weinberg equilibrium test in the UKBB dataset; therefore, PGS and rs1229984 can be used together to identify those with a low risk of AUD. It is noteworthy that the results of the bottom 10% and 5% were similar; therefore, these findings suggest that we can also use the bottom 10% to identify large numbers of low-risk individuals to help understand the etiologies of AUD and develop tailored prevention and treatment strategies.

### Limitations

This study has several limitations. First, both AOU and IB are not representative of general populations of European ancestry, and the prevalence of AUD in the AOU cohort is much lower than in general populations. More datasets are needed to further test the PGS generalizability. Second, familial AUD may have different AUD-associated genes; therefore, using the COGA and OZALC datasets in the screening stage could include familial AUD genes but may miss those from nonfamilial AUD cases, resulting in decreased PGS generalizability. Third, we excluded individuals with other substance use disorders from the controls in the screening datasets. Although this increased statistical power, the estimated effect sizes may be biased and thus could also decrease the PGS generalizability. Fourth, although the same SNVs were used in calculating PGS, different studies used different genotyping platforms, which have a substantial effect on PGS distributions, making it challenging to determine uniform thresholds to define high or low risks in different studies. Fifth, we did not perform PGS analysis in populations of non-European ancestry because the sample sizes were small. Furthermore, for admixed populations such as those of African and Latinx ancestry, different individuals may have different proportions of admixtures, resulting in different LD patterns and carrying different disease-associated SNVs. Large numbers of samples and novel and robust PGS methods for populations of non-European ancestry are critically needed.

Although the PGS could potentially be used to assess AUD risk, it is important to emphasize that the PGS is not intended for diagnosis or prognosis. Accurate diagnosis and prognosis rely on symptom-based assessments. We also emphasize that having a high or low PGS does not guarantee that an individual will or will not develop AUD in the future. Furthermore, the PGS does not consider nongenetic factors that are known to contribute to both AUD risk and resilience. The PGS must thus be interpreted with caution and should never be used to discriminate, to stigmatize, or to deny access to insurance and prevention or treatment programs.

## Conclusions

In this genetic association study, we assessed the promising application of PGS calculated using concordant SNVs to evaluate AUD risk. Future studies will aim to further improve the generalizability of PGS by testing datasets with similar AUD prevalence as in general populations. We will also develop genotyping arrays and methods to eliminate genotyping platform effects and thus allow uniform thresholds for the determination of high or low risk in populations of European ancestry. Additionally, we will develop a PGS that can be used to estimate AUD risk in populations of non-European ancestry.

## Notes

Supplement 1. **eMethods. **Screening and Testing Data Processing and Imputation **eAppendix. **Study Funding and Datasets Used in the Study
eReferences

Supplement 2. **eTable 1.** Polygenic Score (PGS) Analysis Results in the Screening Stage **eTable 2.** The 9154 Single-Nucleotide Variants and Their Weights **eTable 3.** Polygenic Score (PGS) Analysis Results After Excluding rs1229984 T Allele Carriers

Supplement 3.
Data Sharing Statement
