# Supplementary material for: Alcohol Use Disorder Polygenic Score Compared With Family History and ADH1B
Source: JAMA Netw Open. 2024 Dec 30;7(12):e2452705. doi: 10.1001/jamanetworkopen.2024.52705 (PMC11686414; doi:10.1001/jamanetworkopen.2024.52705)
Supplement: Supplement 1. — eMethods. Screening and Testing Data Processing and Imputation eAppendix. Study Funding and Datasets Used in the Study eReferences [file jamanetwopen-e2452705-s001.pdf]

Supplementary Online Content

Lai D, Zhang M, Abreu M, et al. Alcohol use disorder polygenic score compared with family history and *ADH1B*. *JAMA Netw Open*. 2024;7(12):e2452705.
doi:10.1001/jamanetworkopen.2024.52705

eMethods. Screening and Testing Data Processing and Imputation
eAppendix. Study Funding and Datasets Used in the Study
eReferences

This supplementary material has been provided by the authors to give readers additional information about their work.

© 2024 Lai D et al. *JAMA Network Open.*

eMethods. Screening and Testing Data Processing and Imputation

Detailed data processing information for COGA, SAGE, OZALC, and IB was described previously[1-4]. For COGA, SAGE, and OZALC, SNVs with genotyping rate <95%, minor allele frequency (MAF) <3%, or Hardy-Weinberg equilibrium (HWE) P-value <0.0001 were excluded. Samples with inconsistent genetically determined sex (based on X and Y chromosome data) and reported sex were excluded. Mendelian error checking was performed using PedCheck[5] and inconsistencies were set as missing. Genotype data were phased using SHAPEIT2[6] then imputed using Minimac3[7]. For IB, SNVs with genotyping rate <95%, MAF <1%, or HWE P-value <0.0001 were excluded. We used lower MAF in IB as they were genotyped using newer arrays with higher coverages. Genotype data were imputed by using the Michigan Imputation Server[7]. The 1000 Genomes Project data[8] was used as the reference panel for all imputations. Imputed SNVs with R^2 <0.30 or MAF <1% were excluded. Genotype dosages were used in calculating PGS to account for imputation uncertainty and to retain more SNVs.

COGA, SAGE, OZALC, and IB data were combined to detect possible overlapping samples and confirm the reported family relationships. We used a set of high quality (i.e., genotyping rate >98%, MAF >10%, and HWE P-value >0.0001) and independent (linkage disequilibrium (LD) r^2 <0.5) SNVs to calculate identity-by-descent using PLINK[9,10]. For overlapped samples, only those from the dataset with the most detailed phenotypic information were retained. Reported family relationships were updated if necessary. The same set of high-quality, independent SNVs was also used to calculate principal components (PCs) of population stratification. All datasets were combined with the 1000 Genomes Project data[8], and PCs were calculated using PLINK[10]. Based on the first two PCs, individuals clustered with the EA samples from the 1000 Genomes Project were included.

© 2024 Lai D et al. *JAMA Network Open.*

AOU data were whole genome sequenced. Due to the data use policy, all data processing and analyses were performed at the AOU workbench. We used AOU PLINK files that have population-specific allele frequency >1% or population-specific allele count >100 in any ancestry subpopulation. Missing genotypes were imputed as the expected genotypes based on allele frequencies using PLINK[9,10]. Only genetically determined EA samples were included in the analysis.

eAppendix. Study Funding and Datasets Used in the Study

The Collaborative Study on the Genetics of Alcoholism (COGA), Principal Investigators B. Porjesz, V. Hesselbrock, T. Foroud; Scientific Director, A. Agrawal; Translational Director, D. Dick, includes ten different centers: University of Connecticut (V. Hesselbrock); Indiana University (H.J. Edenberg, T. Foroud, Y. Liu, M.H. Plawecki); University of Iowa Carver College of Medicine (S. Kuperman, J. Kramer); SUNY Downstate Health Sciences University (B. Porjesz, J. Meyers, C. Kamarajan, A. Pandey); Washington University in St. Louis (L. Bierut, J. Rice, K. Bucholz, A. Agrawal); University of California at San Diego (M. Schuckit); Rutgers University (J. Tischfield, D. Dick, R. Hart, J. Salvatore); The Children's Hospital of Philadelphia, University of Pennsylvania (L. Almasy); Icahn School of Medicine at Mount Sinai (A. Goate, P. Slesinger); and Howard University (D. Scott). Other COGA collaborators include: L. Bauer (University of Connecticut); J. Nurnberger Jr., L. Wetherill, X., Xuei, D. Lai, S. O'Connor, (Indiana University); G. Chan (University of Iowa; University of Connecticut); D.B. Chorlian, J. Zhang, P. Barr, S. Kinreich, G. Pandey (SUNY Downstate); N. Mullins (Icahn School of Medicine at Mount Sinai); A. Anokhin, S. Hartz, E. Johnson, V. McCutcheon, S. Saccone (Washington University); J. Moore, F. Aliev, Z. Pang, S. Kuo (Rutgers University); A.

© 2024 Lai D et al. *JAMA Network Open.*

Merikangas (The Children's Hospital of Philadelphia and University of Pennsylvania); H. Chin and A. Parsian are the NIAAA Staff Collaborators. We continue to be inspired by our memories of Henri Begleiter and Theodore Reich, founding PI and Co-PI of COGA, and also owe a debt of gratitude to other past organizers of COGA, including Ting- Kai Li, P. Michael Conneally, Raymond Crowe, and Wendy Reich, for their critical contributions. This national collaborative study is supported by NIH Grant U10AA008401 from the National Institute on Alcohol Abuse and Alcoholism (NIAAA) and the National Institute on Drug Abuse (NIDA).

The All of Us Research Program is supported by the National Institutes of Health, Office of the Director: Regional Medical Centers: 1 OT2 OD026549; 1 OT2 OD026554; 1 OT2 OD026557; 1 OT2 OD026556; 1 OT2 OD026550; 1 OT2 OD 026552; 1 OT2 OD026553; 1 OT2 OD026548; 1 OT2 OD026551; 1 OT2 OD026555; IAA #: AOD 16037; Federally Qualified Health Centers: HHSN 263201600085U; Data and Research Center: 5 U2C OD023196; Biobank: 1 U24 OD023121; The Participant Center: U24 OD023176; Participant Technology Systems Center: 1 U24 OD023163; Communications and Engagement: 3 OT2 OD023205; 3 OT2 OD023206; and Community Partners: 1 OT2 OD025277; 3 OT2 OD025315; 1 OT2 OD025337; 1 OT2 OD025276. In addition, the All of Us Research Program would not be possible without the partnership of its participants.

We want to acknowledge the participants and investigators of the FinnGen study. The FinnGen study is a large-scale genomics initiative that has analyzed over 500,000 Finnish biobank samples and correlated genetic variation with health data to understand disease mechanisms and predispositions. The project is a collaboration between research organizations and biobanks within Finland and international industry partners.

© 2024 Lai D et al. *JAMA Network Open.*

This study was made possible, in part, with support from the Indiana Clinical and Translational Sciences Institute funded, in part by Award Number UL1TR002529 from the National Institutes of Health, National Center for Advancing Translational Sciences, Clinical and Translational Sciences Award, and the National Center for Research Resources, Construction grant number RR020128 and the Lilly Endowment. The content is solely the responsibility of the authors and does not necessarily represent the official views of the National Institutes of Health.

The authors acknowledge the Indiana University Pervasive Technology Institute for providing [HPC (Big Red II, Karst, Carbonate), visualization, database, storage, or consulting] resources that have contributed to the research results reported within this paper.

eReferences

1. Lai D, Wetherill L, Kapoor M, et al. Genome-wide association studies of the self-rating of effects of ethanol (SRE). *Addict Biol*. Mar 2020;25(2):e12800. doi:10.1111/adb.12800
2. Lai D, Wetherill L, Bertelsen S, et al. Genome-wide association studies of alcohol dependence, DSM-IV criterion count and individual criteria. *Genes Brain Behav*. Jul 2019;18(6):e12579. doi:10.1111/gbb.12579
3. Lai D, Kuo SI, Wetherill L, et al. Associations between alcohol use disorder polygenic score and remission in participants from high-risk families and the Indiana Biobank. *Alcohol Clin Exp Res (Hoboken)*. Dec 6 2023;doi:10.1111/acer.15239
4. Lai D, Schwantes-An TH, Abreu M, et al. Gene-based polygenic risk scores analysis of alcohol use disorder in African Americans. *Transl Psychiatry*. Jul 5 2022;12(1):266. doi:10.1038/s41398-022-02029-2
5. O'Connell JR, Weeks DE. PedCheck: a program for identification of genotype incompatibilities in linkage analysis. *Am J Hum Genet*. Jul 1998;63(1):259-66. doi:10.1086/301904
6. Delaneau O, Zagury JF, Marchini J. Improved whole-chromosome phasing for disease and population genetic studies. *Nat Methods*. Jan 2013;10(1):5-6. doi:10.1038/nmeth.2307
7. Das S, Forer L, Schonherr S, et al. Next-generation genotype imputation service and methods. *Nat Genet*. Oct 2016;48(10):1284-1287. doi:10.1038/ng.3656
8. Auton A, Abecasis GR, Altshuler DM, et al. A global reference for human genetic variation. *Nature*. 2015/10/01 2015;526(7571):68-74. doi:10.1038/nature15393
9. Purcell S, Neale B, Todd-Brown K, et al. PLINK: a tool set for whole-genome association and population-based linkage analyses. *Am J Hum Genet*. Sep 2007;81(3):559-75. doi:AJHG44631 [pii] 10.1086/519795
10. Chang CC, Chow CC, Tellier LC, Vattikuti S, Purcell SM, Lee JJ. Second-generation PLINK: rising to the challenge of larger and richer datasets. *Gigascience*. 2015;4:7. doi:10.1186/s13742-015-0047-8

© 2024 Lai D et al. *JAMA Network Open.*
