# Supplementary material for: Alcohol Use Disorder Polygenic Score Compared With Family History and ADH1B
Source: JAMA Netw Open. 2024 Dec 30;7(12):e2452705. doi: 10.1001/jamanetworkopen.2024.52705 (PMC11686414; doi:10.1001/jamanetworkopen.2024.52705)
Supplement: Supplement 3. — Data Sharing Statement [file jamanetwopen-e2452705-s003.pdf]

Data Sharing Statement

Lai. Alcohol Use Disorder Polygenic Score Compared With Family History and ADH1B. *JAMA Netw Open*. Published December 30, 2024. doi:10.1001/jamanetworkopen.2024.52705

Data
Data available: No

Additional Information
Explanation for why data not available: Individual patient data will not be shared due to data sharing policy. All results will be shared.
